# Epigenetic Control of Cell Division and Cell Differentiation in the Root Apex

**DOI:** 10.3389/fpls.2015.01178

**Published:** 2015-12-24

**Authors:** Hirotomo Takatsuka, Masaaki Umeda

**Affiliations:** ^1^Graduate School of Biological Sciences, Nara Institute of Science and TechnologyNara, Japan; ^2^Japan Science and Technology, Core Research for Evolutional Science and Technology AgencyIkoma, Japan

**Keywords:** epigenetics, root, *Arabidopsis*, histone modification, DNA methylation, histone chaperone

## Abstract

Epigenetics is defined as heritable changes in gene expression and genome integrity that are accompanied by no alteration in DNA sequence. Throughout plant life cycle, many processes, including genome imprinting, stress responses, and cellular differentiation, are known to be determined by epigenetic regulation. The root apex is also considered to be under the control of epigenetic regulation for optimal growth under variable environments. Recent reports reveal that epigenetic control is especially important in the stem cell niche and the meristematic zone where both cell production and cell specification occur. DNA methylation, histone methylation, and histone acetylation are well-known epigenetic modifications, and each epigenetic modification has distinct roles in roots. Here, we review the updated findings that demonstrate the significance of epigenetic regulation in root apex of *Arabidopsis*.

## Epigenetic Modifications in Plants

The word “Epigenetics” was coined by C. H. Waddington in 1942 as a combination of the words “epigenesis” and “genetics”. In current parlance, epigenetics is defined as the study of mitotically and/or meiotically heritable changes in gene function that cannot be explained by changes in DNA sequence (Riggs et al., 1996). In this paper, we highlight the key events that play a significant role in plant epigenetics, such as DNA methylation, histone modification, and histone chaperones.

DNA methylation is a process by which methyl groups are attached to DNA. It is involved in various biological processes in plants, such as transcriptional repression of transposable elements (TEs) and repetitive sequences, and genomic imprinting. DNA methylation occurs in both the promoter and the gene body. DNA methylation at the promoter and gene body has different effects on the gene expression. DNA methylation in promoter regions usually represses gene expression (Bell and Felsenfeld, 2000; Suzuki and Bird, 2008). In contrast, modest methylation in gene body can promote gene expression, and extremely low or high levels of DNA methylation lead to lower gene expression (Takuno and Gaut, 2012, 2013). In plants, cytosine in three sequence contexts, CG, CHG, and CHH (H = A, C, or T), can be methylated [for a review, see Henderson and Jacobsen (2007)]. DNA methylation is established *de novo* by *DRM2*, and maintained by *DRM2*, *MET1*, and *CMT3*, all of which encode DNA methyltransferases [for a review, see Matzke et al. (2007)]. Demethylation enzymes, such as DME and ROS1, actively remove the methyl group attached to DNA (Choi et al., 2002; Gong et al., 2002).

Histones are highly conserved proteins in eukaryotes that package DNA into structural units called nucleosomes, which provide sites for two copies each of histone H2A, H2B, H3, and H4 proteins. Histone tails are the sites for covalent modifications, such as acetylation, methylation, phosphorylation, ubiquitination, SUMOylation, ribosylation, and biotinylation (Berger, 2007). Amino acids on N-peptide tails of H3 and H4 protrude from nucleosomes and are easily modified. Among various kinds of histone modifications, acetylation, and methylation of H3 and H4 are the best characterized in plants. Gene expression is generally upregulated by acetylation, mono-, di-, or tri-methylation of histone H3 Lysine 4 (H3K4me1, H3K4me2, or H3K4me3), and di- or tri-methylation of histone H3 Lysine 36 (H3K36me2 or H3K36me3), while it is repressed by dimethylation of histone H3 Lysine 9 (H3K9me2) and trimethylation of histone H3 Lysine 27 (H3K27me3) [for a review, see Chen et al. (2010)]. Histone modifications have essential roles in plant development, such as seed development, vegetative growth, floral induction, and flower morphogenesis [for a review, see Wagner (2003)].

Chromatin structures are controlled not only at the level of histone modification, but also of histone dynamics, which are regulated by histone chaperones. Histone chaperones are highly conserved through evolution. The chaperone anti-silencing function 1 (ASF1) binds H3–H4 dimers (English et al., 2006; Natsume et al., 2007) in the cytoplasm, and is involved in histone import into the nucleus (Campos et al., 2010). ASF1 then transfers histones to chaperone complexes involved in nucleosome assembly. The *Arabidopsis* genome contains two genes encoding ASF1 orthologs, *ASF1A* and *ASF1B* (Zhu et al., 2011). In mammals, two distinct pathways control deposition of either the canonical histone H3.1 or the variant H3.3. Chromatin Assembly Factor 1 (CAF-1), consisting of three subunits p150, p60, and p48, promotes histone deposition in a DNA synthesis-dependent manner (Smith and Stillman, 1989; Gaillard et al., 1996). CAF-1 specifically deposits H3.1 interacting with ASF1 (Tyler et al., 2001; Tagami et al., 2004). In *Arabidopsis*, p150, p60, and p48 are referred to as FASCIATA1 (FAS1), FASCIATA2 (FAS2), and MULTICOPY SUPPRESSOR OF IRA1 (MSI1), respectively (Kaya et al., 2001). Plants with mutant copies of *ASF1*s, *FAS1*, and *FAS2* show dramatic and pleiotropic abnormalities during their life cycle, demonstrating the essential roles of histone chaperones in plant development. On the other hand, deposition of histone H3.3 is promoted by Histone Regulator complex (the HIR complex), independent of DNA synthesis and throughout the entire cell cycle (Ray-Gallet et al., 2002; Tagami et al., 2004). HIR complex is constituted by HISTONE REGULATOR A (HIRA), UBINUCLEIN (UBN), and CAlCINEURIN BINDING PROTEIN 1 (CABIN1). In *Arabidopsis*, loss-of-function mutants of *HIRA* display reduced fertility (Duc et al., 2015). Although other types of histone chaperones have been identified in mammals [for a review, see Filipescu et al. (2014)], their orthologs have not been identified in plants thus far.

## Four Distinct Zones in Roots

**FIGURE 1:**
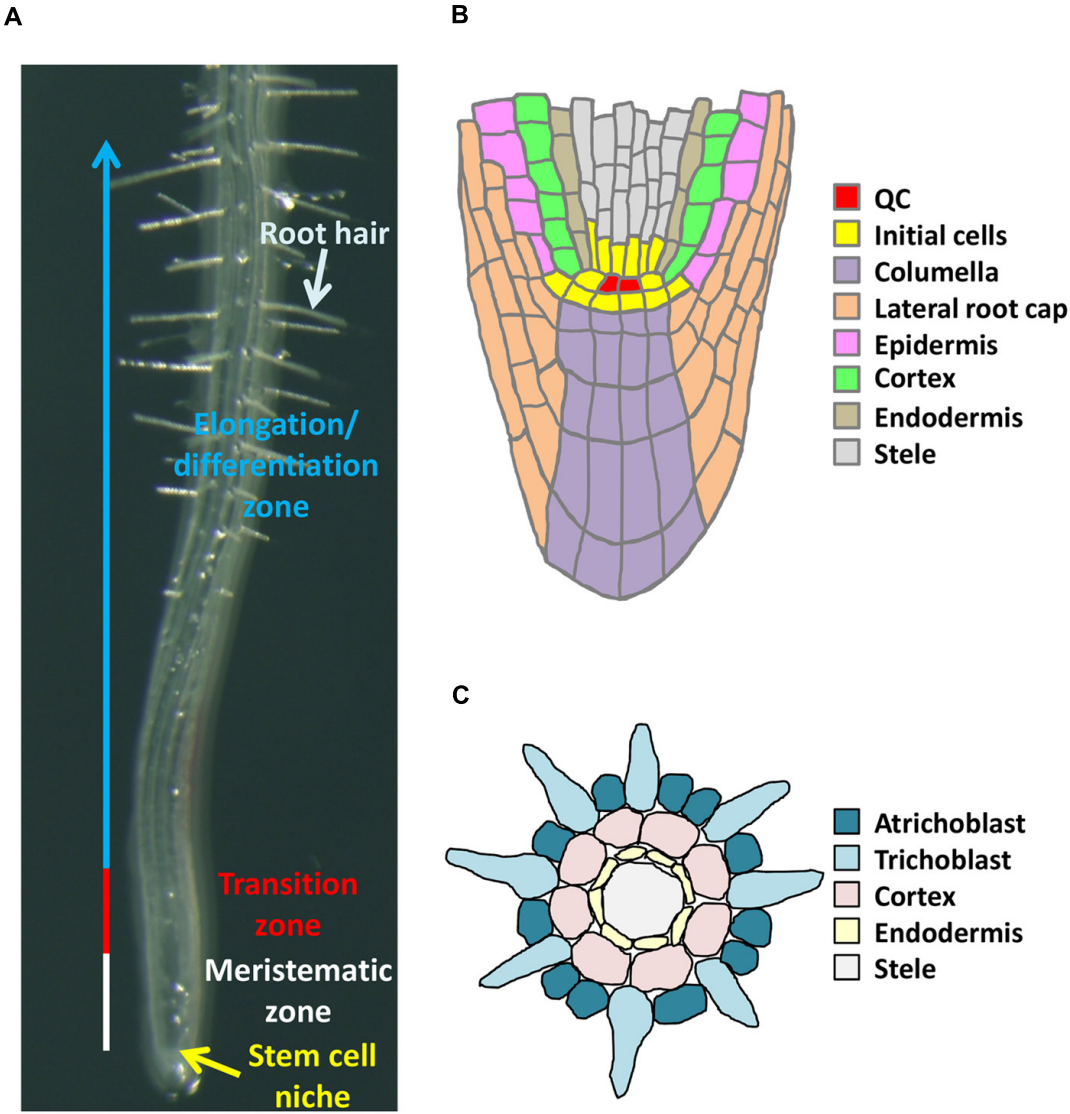
***Arabidopsis* root structure.** **(A)** Four distinct zones. **(B)** Organization of cells in the root tip. **(C)** Transverse section of a root at an early stage of root hair formation.

Roots of *Arabidopsis* are composed of four distinct zones; namely, the stem cell niche (SCN), the meristematic zone (MZ), the transition zone (TZ), and the elongation/differentiation zone (EDZ; **Figure 1A**) (Takatsuka and Umeda, 2014). The SCN is important to orchestrate the fine balance of stem cell maintenance and supply of differentiating daughter cells. The stem cells surrounding the approximately four quiescent center (QC) cells give rise to each cell lineage, such as columella, lateral root cap, epidermis, cortex, endodermis, and provascular tissues (**Figure 1B**). In the MZ, cells generated from the SCN continue to divide a few times, contributing to root growth through increasing cell number. After leaving the MZ, cells enter the TZ and initiate endoreplication. In the TZ, cells slowly elongate in the direction of both width and length. Although the significance of TZ is not well understood, recent reports shed light on endoreplication-mediated cell elongation in the TZ (Adachi et al., 2011; Takahashi et al., 2013; Takatsuka and Umeda, 2014). The EDZ mainly has two important roles; one is to elongate roots by increasing each cell’s volume, and the other one is to develop root hairs for effective uptake of water and mineral nutrients from the soil (**Figures 1A,C**). Below we summarize the role of epigenetic control in establishment and maintenance of each root zone.

## Specification of the Stem Cell Niche and Control of Cell Division in the Meristematic Zone

**FIGURE 2:**
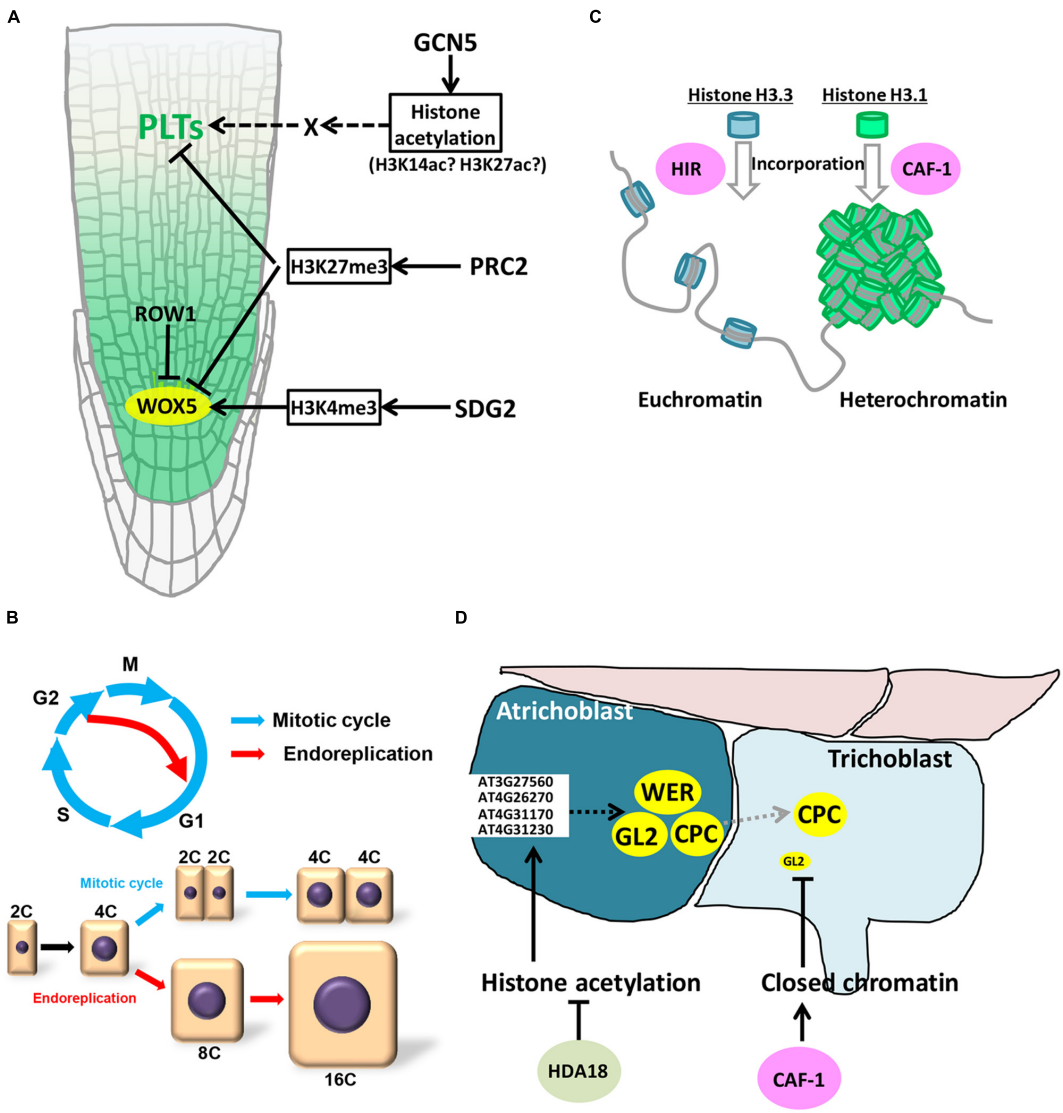
**Epigenetic control in roots.** **(A)** Epigenetic control of *WOX5* and *PLT*s expression. **(B)** Endoreplication. **(C)** HIR- and CAF-1-mediated formation of euchromatin and heterochromatin. **(D)** Roles of CAF-1 and histone acetylation in root hair development.

The SCN consists of the QC and surrounding initial cells. For indeterminate growth of roots, it is indispensable to maintain QC cells intact (**Figure 1A**). *PLETHORA*s (*PLT*s) and *WUSCHEL RELATED HOMEOBOX 5* (*WOX5*) play crucial roles in specifying QC cells in *Arabidopsis* (Aida et al., 2004; Sarkar et al., 2007). Both genes encode transcriptional factors; PLTs (PLT1 to PLT6) are AP2 domain-containing transcription factors, among which PLT1 and PLT2 play important roles in roots, and WOX5 is a WUSCHEL-related homeobox transcription factor. QC misspecification and resultant dysfunction of initial cells are observed in *plt* and *wox5* mutants, indicating essential functions of these genes in SCN maintenance (Aida et al., 2004; Sarkar et al., 2007). *WOX5* expression is confined to the QC (Sarkar et al., 2007) (**Figure 2A**), while *PLT*s are expressed over the MZ, forming a gradient with a maximum near the root tip (**Figure 2A**). This gradient has been reported to be prerequisite for the zonation of *Arabidopsis* roots (Galinha et al., 2007; Mähönen et al., 2014). Namely, high levels of *PLT* expression promote stem cell maintenance; intermediate levels of expression enhance mitotic activity in the MZ; *PLT* expression under the threshold level is required for cell differentiation and cell elongation in the EDZ (Galinha et al., 2007; Mähönen et al., 2014). Some reports demonstrate that expression of *PLT*s and *WOX5* is under the control of epigenetic modifiers.

### Histone Acetylation

Histone acetylation catalyzed by histone acetyltranferases (HATs) is generally correlated with active transcription. *Arabidopsis* has four HAT families: GNAT (GCN5-related N-terminal acetyltransferases), MYST (MOZ, Ybt2, Sas2, Tip60-like), p300/CREB-binding protein (CBP), and TAFII250 families (Pandey et al., 2002). The GCN5 ortholog of the GNAT family is the best-characterized HAT in *Arabidopsis* (Pandey et al., 2002; Chen and Tian, 2007). Similar to yeast and mammals, *Arabidopsis* GCN5 interacts with ADA2a and ADA2b, and acetylates histone H3 (Stockinger et al., 2001; Mao et al., 2006; Earley et al., 2007). Histone H3 lysine 14 (H3K14) and H3K27 acetylation marks are reduced at defined loci in *gcn5* mutants (Benhamed et al., 2006). Globally, the GCN5 complex regulates a subset of genes (∼5%) in the *Arabidopsis* genome (Vlachonasios et al., 2003; Benhamed et al., 2008). In the mutants of both *GCN5* (also called *HAG1*) and *ADA2b*, expression of *PLT1* and *PLT2* was dramatically reduced, resulting in perturbed QC specification and a smaller meristem (Kornet and Scheres, 2009) (**Figure 2A**). Overexpression of *PLT2* partially rescued the reduced meristem size of *gcn5* mutants, suggesting that *GCN5* acts in the *PLT* pathway (Kornet and Scheres, 2009) (**Figure 2A**). This indicates that the histone acetylation controlling *PLT* expression is essential for root zonation. However, it is still ambiguous whether the expression of *PLT* genes is directly regulated by the GCN5 complex. Anzola et al. (2010) showed that H3K9ac/K14ac was detected on the *PLT2* promoter, although the acetylation level was sustained in *ada2b* mutants, suggesting a possibility of GCN5-independent histone acetylation. ChIP-chip analysis with anti-GCN5 antibodies also revealed that GCN5 did not associate with *PLT* genes (Benhamed et al., 2008) (**Figure 2A**). In support of this idea, both GCN5 and ADA2b are expressed ubiquitously in the MZ, whereas *PLT* expression is higher in the SCN and lower in the MZ, indicating that the gradient of *PLT* expression is regulated by factors downstream of *GCN5*. Since auxin is known to accumulate in the SCN and is involved in *PLT* expression, the GCN5 complex may control expression of some auxin-related factors, such as AUX/IAAs and ARFs, which then display the *PLT* gradient in the root tip. The recent report demonstrated that, under auxin-enriched conditions, bZIP11-related basic leucine zipper (bZIP) transcription factors bind the promoters of auxin-responsive genes and recruit histone acetylation machinery by interacting with ADA2b, thereby boosting the expression of auxin-responsive genes (Weiste and Dröge-Laser, 2014). Such a link between the GCN5 complex and auxin-related factors may explain consequent formation of the *PLT* gradient in the root tip.

### Histone Methylation

The evolutionarily conserved Polycomb group (PcG) and trithorax group (trxG) proteins are central regulators of cell identity, maintaining the balance between cell proliferation and cell differentiation (Schuettengruber and Cavalli, 2009). PcG proteins form multimeric complexes, which repress transcription of target genes. In *Arabidopsis*, PcG proteins are incorporated into two complexes: Polycomb Repressive Complex1 (PRC1) and Polycomb Repressive Complex2 (PRC2). PRC2 is the best-studied PcG complex that catalyzes trimethylation of histone H3 lysine 27 (H3K27me3). The core component of PRC2 is composed of the SET-domain H3K27 methyltransferase, CURLY LEAF (CLF), and SWINGER (SWN), the latter of which has a partially overlapping function with CLF. In the *Arabidopsis* endosperm, MEDEA (MEA) functions as a H3K27 methyltransferase. Some proteins, such as EMBRYONIC FLOWER2 (EMF2), assist methyltransferases as a component of PRC2. It has been reported that, in *Arabidopsis*, around 4400 genes are modified by H3K27me3 (Zhang et al., 2007). Hence the loss of H3K27me3 in the mutant of PRC2 complex causes multiple developmental defects, such as in leaf and endosperm formation, in transition from embryo to seedlings, and in callus regeneration. Recently, Aichinger et al. (2011) reported the role of PRC2 in root development. Loss of CLF function increased meristematic activity, leading to larger MZ and longer roots. Moreover, additional undifferentiated cell layers were formed in columella initials in the *clf* mutant, demonstrating that the mitotic activity is elevated not only in the MZ but also in initial cells in the SCN. In agreement with this observation, *WOX5* expression was upregulated in the mutant, and ChIP-PCR analysis revealed that *WOX5* and other important regulators of the SCN and the MZ, such as *PLT*s and *AGAMOUS-LIKE*s(*AGL*s), are trimethylated at H3K27 (Aichinger et al., 2011) (**Figure 2A**). On the other hand, in the mutant of chromatin remodeling factor *PICKLE* (*PKL*), levels of H3K27me3 are substantially increased, suggesting an antagonistic interaction between PRC2 and PKL in determining the root zonation. However, PKL is ubiquitously expressed throughout the MZ and the SCN, and CLF is highly expressed in the MZ but poorly in the EDZ (Aichinger et al., 2011; Gu et al., 2014). These overlapping expression patterns indicate that root zonation is defined through reciprocal activities, rather than expression patterns, of PRC2 and PKL. Further analyses will reveal how their activities are controlled in distinct zones of roots.

Histone H3 lysine 4 di- and trimethylation variants (H3K4me2 and H3K4me3) are abundant in euchromatin and, in general, are associated with transcriptional activation in eukaryotes. Several *Arabidopsis* SET DOMAIN GROUP (SDG) proteins (ATX1, ATX2, ATXR7, and SDG2) have been shown to exhibit TrxG-like H3K4-methyltransferase activity. Root growth of *atx1* and *sdg2* mutants is severely impaired due to abnormal SCN organization and reduced mitotic activity in the MZ (Yao et al., 2013; Napsucialy-Mendivil et al., 2014). *PLT1* expression is decreased in *sdg2* mutants, suggesting a possibility that SDG2-mediated H3K4me2/3 controls root development via regulating *PLT1* expression (Yao et al., 2013). Both ATX1 and SDG2 are strongly expressed in the MZ, and a gradient of expression, such as that of the *PLT*s, has not been reported so far. Therefore, ATX1 and SDG2, as well as GCN5 and ADA2b as mentioned above, likely contribute to the gradient formation of *PLT* expression in collaboration with other unidentified factors.

As for *WOX5*, it has been recently shown that H3K4me3 on the *WOX5* promoter region has a crucial role in QC-specific expression of *WOX5* (Zhang et al., 2015). ROW1 is a PHD-containing protein with two tandem BRCA1 C-terminal domains and a RING domain, and is expressed in the MZ, but not in the QC (**Figure 2A**). In the MZ, ROW1 binds to H3K4me3 on the *WOX5* promoter to sequester the promoting function of H3K4me3 in transcription. However, in the QC, ROW1 expression is absent, thus H3K4me3 can induce *WOX5* expression (**Figure 2A**). Consistent with these observations, the *row1* mutation causes ectopic expression of *WOX5* in the root tip, leading to a *WOX5* overexpression-like phenotype, such as loss of starch granules in mature columella cells (Sarkar et al., 2007; Zhang et al., 2015).

### CAF-1

In mutants of the CAF-1 components, FAS1 and FAS2, roots grow more slowly than the wild-type due to lower mitotic activity, and SCN organization is perturbed (Leyser and Furner, 1992; Kaya et al., 2001; Ramirez-Parra and Gutierrez, 2007). The expression pattern of *SCARECROW* (*SCR*), a key gene for SCN establishment and radial pattern formation, is not maintained in *fas* mutant roots. However, the relationship between CAF-1 and other SCN-related genes, such as *WOX5* and *PLT*s, remains unknown.

### DNA Methylation

All DNA methyltransferases, except for *DRM1*, are strongly expressed in the root tip of *Arabidopsis* (Jullien et al., 2012). However, to our knowledge, the effects of mutations of DNA methyltransferases on root phenotype have not been reported thus far. Loss of DNA methylation leads to movement of transposons, which frequently generates gene mutations and causes morphological abnormalities. Therefore, it is difficult to distinguish mutant phenotypes caused by global changes in DNA methylation from that of gene mutations induced by transposon insertion.

## Cell Elongation and Differentiation in the Transition and Elongation/Differentiation Zones

After cells undergo a few rounds of mitosis in the MZ, they enter the TZ and start cell elongation (**Figure 1A**). This cell elongation is caused by endoreplication, a specialized mode of the cell cycle in which DNA replication is repeated without mitosis or cytokinesis. As a result, DNA ploidy increases in each elongating cell (**Figure 2B**). It is known that endoreplication is usually accompanied by cell enlargement and cell differentiation, while the underlying mechanisms remain unknown (Inzé and De Veylder, 2006; Kalve et al., 2014). The involvement of epigenetic regulation in endoreplication has been poorly understood, but here we discuss a possible link between epigenetic control and genome stability that is associated with the onset of endoreplication.

After leaving the TZ, cells enter the EDZ where rapid cell elongation and terminal differentiation occur (**Figure 1A**). Although cells produced in the MZ are no more than 10 μm in the longitudinal direction, they reach more than few hundred micrometers in the EDZ. Such a dramatic increase in cell volume, as well as cell division in the MZ, greatly contributes to root growth. Recently one report demonstrated the involvement of epigenetic control in cell elongation in the EDZ.

### CAF-1

As in mammals, histone H3 of plants is classified into two types, H3.1 and H3.3. They differ from each other at positions 31, 41, 87, and 90 (T31Y41H87L90 for H3.3 and A31F41S87A90 for H3.1) (Ingouff and Berger, 2010). In spite of such a high similarity, they have distinct functions in controlling chromatin structure. H3.3 is incorporated into chromatin outside of the S phase, and its deposition is correlated with transcriptionally active genes, suggesting that H3.3 is involved in euchromatin formation (Mito et al., 2005). In contrast, H3.1 is incorporated into chromatin during DNA replication and is enriched in dimethylated H3 Lys9 (H3K9me2), which is associated with gene silencing and heterochromatin formation (**Figure 2C**) (Peters and Schübeler, 2005). As mentioned above, the CAF-1 complex plays an essential role in H3.1 incorporation in a DNA replication-dependent manner. Although plant CAF-1 subunits, FAS1 and FAS2, were originally identified from *Arabidopsis* mutants with abnormal meristem organization, endoreplication was later found to be abnormal in the mutants. Namely, the ploidy level of the *fas1* and *fas2* mutants was higher than that of the wild-type, leading to larger epidermal cells and increased trichome branching on leaves (Exner et al., 2006). In these mutants, expression levels of DNA double-strand break (DSB)-induced genes, such as DNA repair-related genes, were elevated (Schönrock et al., 2006; Ramirez-Parra and Gutierrez, 2007). Moreover, Endo et al. (2006) observed a small but significant increase in the extent of DSBs in the *fas* mutants as compared to the wild-type. Since it was reported that DSBs promote an early onset of endoreplication by inducing G2/M arrest (Adachi et al., 2011), lowered genome stability might result in enhanced endoreplication in the *fas* mutants. This suggests a possibility that CAF-1-mediated H3.1 incorporation is engaged in maintaining genome stability, thus protecting the genome from DNA damage.

The *fas1* and *fas2* mutants have a shorter root meristem (Kaya et al., 2001), indicating a possibility of an early transition from mitosis to endoreplication. If this is the case, CAF-1 may also be involved in the control of genome stability in roots. However, *fas1* and *fas2* mutants exhibit reduced mitotic activity (Ramirez-Parra and Gutierrez, 2007); thus it is also likely that the early transition to endoreplication is a consequence of lowered cyclin-dependent kinase activities, which inhibit the G2/M progression and promote the onset of endoreplication (De Veylder et al., 2011). On the other hand, Napsucialy-Mendivil et al. (2014) reported that in the *atx1-1* mutant, the root meristem size is reduced, while the transition to cell elongation is suppressed. This suggests another possibility that *fas1* and *fas2* mutants have impaired cell division in the MZ, but no defect in endoreplication. Further studies will reveal how CAF-1 is associated with the control of the root meristem size, and whether CAF-1 is actively involved in the control of genome stability in the MZ, where endoreplication is suppressed.

### Histone Acetylation

Rapid cell growth in the EDZ requires cell wall rearrangement, which is controlled by cell wall-related proteins like xyloglucan endotransglucosylase (XET), endo-1,4-β-D-endoglucanase (EGase), expansins (EXP), and the plasma membrane proton pump (PM-H^+^-ATPase, MHA) (Geilfus et al., 2011). In maize, high salinity stress makes roots swollen possibly due to up-regulation of cell wall-related genes (Li et al., 2014). Expression of *ZmEXPB2* and *ZmXET1* is regulated by H3K9 acetylation, and histone acetyltransferase (HAT) genes *ZmHATB* and *ZmEXPB2* are highly expressed under high salinity conditions (Li et al., 2014). Therefore, it is likely that HAT-mediated acetylation of H3K9 is required for induction of *ZmEXPB2* and *ZmXET1* in the EDZ, and that salinity stress up-regulates the expression of *ZmHATB* and *ZmGCN5*, thus enhancing expression of *ZmEXPB2* and *ZmXET1* and leading to cell enlargement and resultant root swelling.

## Root Hair Being Specified in the Meristematic Zone and Developed in the Elongation/Differentiation Zone

Recent reports showed that epigenetic regulation is required not only for determination of differentiation state along the apical-basal axis of roots but also for cell specification. Root epidermal cells consist of two cell types, trichoblast and atrichoblast, in *Arabidopsis*. The trichoblast lineage is in contact with two cortex cells and produces root hairs, whereas atrichoblast in contact with a single cortex cell never forms root hairs (**Figure 1C**). This indicates that positional cues are essential to trigger root hair development. Previous studies showed that position-dependent cellular pattern of epidermal cells is determined by interactions of six patterning genes; *CAPRICE* (*CPC*), *ENHANCER OF TRY AND CPC 1* (*ETC1*), *GLABRA 2* (*GL2*), *GLABRA 3* (*GL3*), *TRANSPARENT TESTA GLABRA 1* (*TTG1*), and *WEREWOLF* (*WER*) [for a review, see Schiefelbein et al. (2014)].

### CAF-1

*GL2* is expressed in atrichoblasts, but not in trichoblasts, and functions as a critical determinant specifying atrichoblast (**Figure 2D**). Indeed, ectopic root hairs are formed from the atrichoblast linage in the *gl2* mutant due to misspecification of atrichoblasts. Costa and Shaw (2006) demonstrated that atrichoblast-specific expression of *GL2* is controlled by CAF-1 complex (**Figure 2D**). The *GL2* locus in the *Arabidopsis* genome is more condensed in trichoblasts as compared to atrichoblasts, resulting in higher accessibility of transcription machineries onto the promoter in atrichoblasts. In the *fas2* mutant, *GL2* is ubiquitously expressed in both trichoblasts and atrichoblasts, resulting in a reduced number of root hairs. This suggests that CAF-1-mediated control of chromatin structure is involved in determination of epidermal cell fate in roots (**Figure 2D**). However, it remains unknown how CAF-1 regulates the *GL2* locus specifically in trichoblasts. Based on the previous report (Kaya et al., 2001) and the *Arabidopsis* expression database (http://bar.utoronto.ca/efp/cgi-bin/efpWeb.cgi), it is unlikely that the *FAS2* expression is specific to the trichoblast. Identifying mechanisms that control the CAF-1 activity in particular cell types will help discover a new regulatory system of cell differentiation during root development.

### Histone Acetylation

Xu et al. (2005) found that treatment of *Arabidopsis* roots with trichostatin A (TSA), a specific inhibitor of histone deacetylase (HDAC), induces hair cell development ectopically in the atrichoblast lineage (**Figure 2D**). They showed that TSA treatment altered the expression of *CPC*, *GL2*, and *WER* in the atrichoblast, resulting in ectopic root hair development (Xu et al., 2005). Among the 18 *HDAC* genes of *Arabidopsis* (Hollender and Liu, 2008), *HDA18* is specifically required for proper root hair development; in the *hda18* mutant, the root hair was occasionally formed in the atrichoblast lineage, reminiscent of TSA-treated roots (Xu et al., 2005). Liu et al. (2013) reported that expression of some kinase genes (At3G27560, At4G26270, At4G31170, and At4G31230) is directly regulated by HDA18, and their mutants and overexpressors displayed root hair phenotypes. Therefore, it is probable that HDA18 controls the expression of *CPC*, *GL2*, and *WER* via these kinases in atrichoblast, suppressing root hair development in this cell lineage (Liu et al., 2013).

## Summary and Future Perspectives

Many reports have demonstrated that epigenetic regulation plays an indispensable role in zonation of roots. However, as described in this article, epigenetic regulators are not expressed in a gradient or in a zone-specific manner. Rather, in many cases, they are expressed ubiquitously in roots, indicating that one epigenetic regulator cannot explain how to make the root zonation. One possibility to explain this issue is that interaction of multiple epigenetic regulators forms the gradient or zone-specific expression of key genes. For example, the *PLT* gradient may be formed as a result of combined action of the GCN5 complex, PRC2 and PKL as described above. In mouse embryonic stem cells, genes with H3K4me2 are generally highly expressed, whereas those with H3K27me3 are poorly expressed. In fact, many genes are bivalently marked with both H3K4me2 and H3K27me3, and their expression is lower as that of the genes with H3K27me3 marks only, demonstrating that the effect of H3K27me3 on gene expression is epistatic to that of H3K4me2 (Bernstein et al., 2006). There are many types of epigenetic modifications also in plants, thus it is probable that complex relationship among various epigenetic factors might be required to establish the root zonation. Another possibility is that unidentified factors regulating the activity of epigenetic regulators are expressed in a gradient or in a zone-specific manner. Although it is poorly understood how the activity of epigenetic regulators, such as histone methyltransferases and histone acetyltransferases, is regulated, ROW1 is a good example showing how epigenetic modifications are controlled by another factor. Further identification of such modifiers of epigenetic regulation will give a mechanistic insight into cell division, cell growth, and cell differentiation in each zone of roots.

## Author Contributions

HT and MU wrote the paper cooperatively.

## Conflict of Interest Statement

The authors declare that the research was conducted in the absence of any commercial or financial relationships that could be construed as a potential conflict of interest.
